# Monocytic THP-1 cells diverge significantly from their primary counterparts: a comparative examination of the chromosomal conformations and transcriptomes

**DOI:** 10.1186/s41065-021-00205-w

**Published:** 2021-11-05

**Authors:** Yulong Liu, Hua Li, Daniel M. Czajkowsky, Zhifeng Shao

**Affiliations:** State Key Laboratory for Oncogenes & Related Genes and Bio-ID Center, School of Biomedical Engineering, Shanghai Jiao Tong University, Shanghai, 200240 China

**Keywords:** Hi-C, Chromatin conformation, Monocytes, Macrophages, Transcriptome

## Abstract

Immortalized cell lines have long been used as model systems to systematically investigate biological processes under controlled and reproducible conditions, providing insights that have greatly advanced cellular biology and medical sciences. Recently, the widely used monocytic leukemia cell line, THP-1, was comprehensively examined to understand mechanistic relationships between the 3D chromatin structure and transcription during the trans-differentiation of monocytes to macrophages. To corroborate these observations in primary cells, we analyze in situ Hi-C and RNA-seq data of human primary monocytes and their differentiated macrophages in comparison to that obtained from the monocytic/macrophagic THP-1 cells. Surprisingly, we find significant differences between the primary cells and the THP-1 cells at all levels of chromatin structure, from loops to topologically associated domains to compartments. Importantly, the compartment-level differences correlate significantly with transcription: those genes that are in A-compartments in the primary cells but are in B-compartments in the THP-1 cells exhibit a higher level of expression in the primary cells than in the THP-1 cells, and vice versa. Overall, the genes in these different compartments are enriched for a wide range of pathways, and, at least in the case of the monocytic cells, their altered expression in certain pathways in the THP-1 cells argues for a less immune cell-like phenotype, suggesting that immortalization or prolonged culturing of THP-1 caused a divergence of these cells from their primary counterparts. It is thus essential to reexamine phenotypic details observed in cell lines with their primary counterparts so as to ensure a proper understanding of functional cell states in vivo.

**Supplementary Information:**

The online version contains supplementary material available at 10.1186/s41065-021-00205-w.

## Background

Immortalized cell lines are one of the pillars of contemporary cell biology, providing essentially unlimited access to experimental material and highly pure cell populations [1, 2]. Their use has unquestionably advanced our understanding of molecular mechanisms underlying biological processes in many areas of biomedical research [3–7]. A notable recent example is the comprehensive examination of the changes in chromatin structure that are associated with the changes in transcription during the monocyte-to-macrophage differentiation in the model monocytic cell line THP-1, which was originally derived from an acute myeloid leukemia patient [3]. One of the critical discoveries in this work is that the transcription factor AP-1, observed to be up-regulated during this transition, plays a fundamental role in both static and dynamic chromosome structure, specifically at the level of loops [8].

In parallel, this monocyte-to-macrophage trans-differentiation process was also studied in primary human monocytes using in situ Hi-C and RNA-seq [9]. Intriguingly, the up-regulation of AP-1 during this transition was not confirmed in these primary cells. This observation raises the question of to what extent do the THP-1 cells and their differentiated macrophages resemble their primary counterparts, a question with broad implications for other immortalized/primary cell pairings as well.

To shed light on this issue, we compared the chromatin structures of the monocytic/macrophagic THP-1 cells and their primary counterparts by reanalyzing these recently available Hi-C data. Unexpectedly, we find that their chromatin structures are dramatically different at all levels of conformation, from loops to topologically associated domains (TADs) to compartments. Further, the differences in structure at the compartment-level correlate significantly with differences in the expression of genes localized within these regions, including those that are upregulated in the primary cells that are associated with typical functions of monocytic immune cells. Thus, overall, these results reveal a divergence of the THP-1 cells during immortalization or prolonged culturing from their primary counterparts, underscoring the requirement to ultimately validate observations obtained from immortalized cultured cells with primary cells for a faithful characterization of functional states in vivo.

## Results and discussion

The recent publication of in situ Hi-C data of the monocytic THP-1 cells and their differentiated macrophage induced by the addition of phorbol myristate acetate (PMA), together with that of the in situ Hi-C study of human primary monocytes and the granulocyte-macrophage colony stimulating factor (GM-CSF)-induced macrophages, provides a unique opportunity to validate the observations obtained from this commonly used immortalized cell line with its primary counterpart.

Following a well-established method [10], we first compared larger-length scale, compartment-level interactions, after first verifying that the A-compartments are generally enriched for active genes and the B-compartments are enriched for inactive genes for each of the four cell types (Supplementary Fig. S1, Supplementary Table 1) [11]. We found that there is a significant difference (20%) in compartment-level interactions between the primary monocytes and monocytic THP-1 cells (Supplementary Fig. S2a), which is consistent with previous analysis [9]. A comparable difference (19%) in compartment-level interactions was also found between the primary, GM-CSF-induced macrophages and the macrophagic THP-1 cells (Supplementary Fig. S2b). Such a difference in compartment-level features is generally considered as evidence of substantially reorganized chromatin [12, 13], similar to what is observed following, for example, differentiation of embryonic stem cells [13]. We note that this difference greatly exceeds the degree of difference that has been found between compartment-level Hi-C data obtained from different laboratories [14], arguing against this as a cause for the observed differences. Thus, these results indicate that these immortalized cells have, in fact, evolved to a fundamentally different chromatin structure from their primary counterparts.

**Fig. 1:**
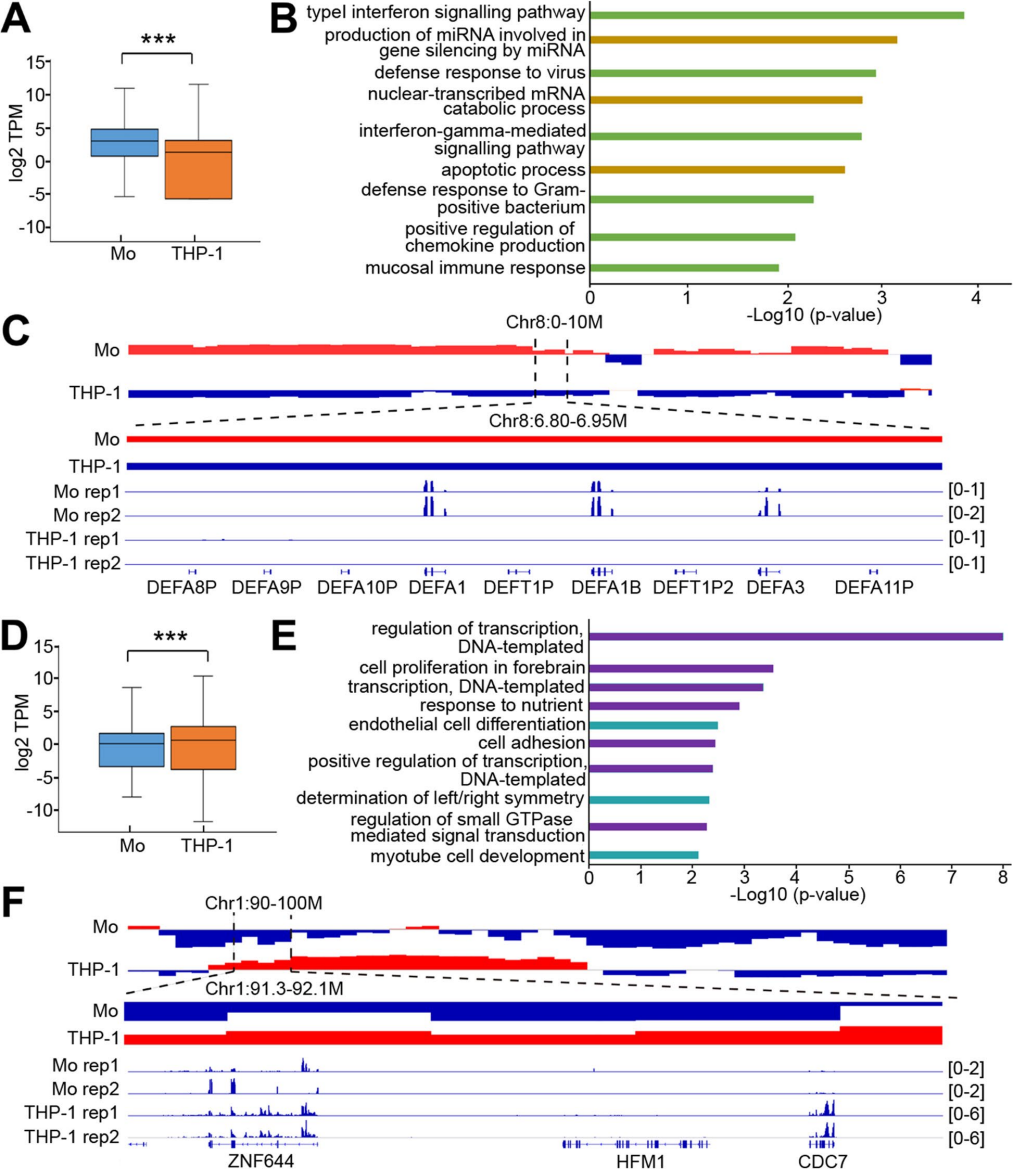
Relationship between differences in compartments and gene expression between primary monocytes and THP-1 cells. **a** Comparison of gene expression levels at regions that switched from A- compartment in the primary monocytes to B-compartment in the monocytic THP-1 cells (“***” represents *p* < 0.001, Wilcoxon rank sum test) TPM refers to transcript per million. **b** Gene ontology analysis of the genes that reside in the compartments described in (**a**). Colored in green are those terms related to typical immune cell functions. **c** Example of immune cell-related genes (DEFA1, DEFA1B, DEFA3) whose changed expression is associated with the different compartments depicted in (**a**). A-compartments are colored in red and B-compartments are colored in blue. **d** Comparison of gene expression levels at regions that switched from B-compartment in primary monocytes to A-compartment in the monocytic THP-1 cells (“***” represents *p* < 0.001, Wilcoxon rank sum test). **e** Gene ontology analysis of the genes reside in the compartments described in (**d**). Colored in purple are those terms related to cell proliferation. **f** Example of a gene that plays a role in the regulation of cell division (CDC7) whose changed expression is associated with the different compartments. A-compartments are colored in red and B-compartments are colored in blue

To determine whether these longer-range structural differences are consequential for gene expression, we compared the level of gene expression within those regions that are in different compartments in the primary and THP-1 cells. For the monocytic-type cells, those genes that are found in the A-compartments in the primary cells but in the B-compartments in the THP-1 cells exhibit a higher level of expression in the primary monocytes than in the THP-1 cells (Fig. 1a, Supplementary Fig. S3, Supplementary Table 1). These genes are enriched for many different gene ontological terms, suggesting that there are many biological pathways affected by these structural differences (Fig. 1b, Supplementary Table 2). However, it is worth noting that many terms are associated with basic immune functions, such as the innate immune response, positive regulation of chemokine production, and defense response to viruses, all important processes in monocytes (Fig. 1b). For example, several genes that encode for human alpha defensins (DEFA1, DEFA1B, and DEFA3), which are involved in host defense [15, 16], all exhibit essentially no expression in the monocytic THP-1 cells where they are in the B-compartment, but have significant expression in primary monocytes where they are in the A-compartment (Fig. 1c, Supplementary Fig. S4). This suggests that the immortalized cells have lost at least some of their original monocytic functions as a consequence of, or in association with, these alterations in chromatin structure. In addition, the genes in the B-compartments in the primary monocytes that have relocated to the A-compartments in the THP-1 cells are found to be upregulated in the THP-1 cells (Fig. 1d, Supplementary Fig. S5, Supplementary Table 1). These THP-1 up-regulated genes are also enriched for many biological pathways, including response to starvation, cell adhesion, and response to nutrients (Fig. 1e, Supplementary Table 2). For example, the gene that encodes for Cell Division Cycle 7-related protein kinase (CDC7), which plays an important role in the regulation of the cell cycle [17], exhibits very little expression in the primary monocytes where they are in the B-compartment, but there is significant expression in monocytic THP-1 cells where they are in the A-compartment (Fig. 1f). It is possible that these genes exhibit greater expression in the THP-1 cells owing to their highly proliferative nature in culture, while for the primary cells, since proliferation is largely absent, these genes are not needed and are thus inactive.

Similarly, when the primary, GM-CSF-induced macrophage cells and the macrophagic THP-1 cells were compared, we also found significant relocation of genes between different compartments (Supplementary Fig. S6a, b). Likewise, the genes in these different compartments are enriched for many biological pathways, suggesting that these structural changes are also associated with differences in the functional states between these cells (Supplementary Fig. S6c, d; Supplementary Table 2). Thus, like the monocytic-type cells, these results suggest that the macrophagic THP-1 cells are also fundamentally different from their primary counterpart.

We next examined for differences in the more local-level interactions of loops and TADs, the latter of which are very highly conserved (> 92%) between many cell types [18, 19]. Since statistically valid comparisons of such high-resolution features requires data at a comparable sequencing depth, we first obtained multiple (5) down-sampled datasets of the original THP-1 data, each at a similar level (90 million reads) as the primary cell datasets (see Materials and Methods). We used TopDom to identify the TADs within each dataset at the highest map resolution possible with this data (40 kb, see Materials and Methods) and calculated the extent to which the TAD locations were identical between different datasets. We found that there was good agreement among the down-sampled monocytic THP-1 datasets (74.1 ± 0.4%), with the less than perfect agreement between these datasets consistent with the lowered sampling depth. In contrast, only 26.1 ± 0.3% of the TADs in the primary monocytes were the same as those in the down-sampled THP-1 datasets, which is significantly different (*p* < 0.001, normality test) from that within the down-sampled datasets (Supplementary Fig. S7a, Supplementary Table 3). Similarly, comparing just the down-sampled macrophagic THP-1 datasets, there was good agreement in the identified TADs (71.2 ± 0.6%). However, only 51.5 ± 0.3% of the TADs in the primary, GM-CSF-induced macrophage cells were the same as those in the down-sampled macrophagic THP-1 cells (Supplementary Fig. S7b, Supplementary Table 3), which is also significantly different (*p* < 0.001, normality test) from the measurements of just the down-sampled datasets. Similar differences, for both monocytic and macrophagic-type cells, were also observed at lower map resolutions as well (Supplementary Fig. S7c, d). This degree of difference is truly exceptional insomuch as, for example, the differentiation of embryonic stem cells was associated with changes in chromatin structure at the compartment-level but with essentially no changes at the TAD-level [13]. This underscores the magnitude of the structural changes in the chromatin that the THP-1 cells have undergone from their presumed original state.

We last compared the differences at the loop-level, using the program Peakachu, which has been shown to robustly identify loops at a 10 kb resolution even for data that is at a lower sequencing depth (down to 30 million reads) than our primary cell data [20]. This method computes the probability that there is a loop in each pixel in the Hi-C map using a machine-learning framework, with bona-fide loops identified as those pixels with probability values above 0.97, a threshold value recommended by the original authors (see Materials and Methods) [20]. With the same consideration as that for the TAD analysis, we first compared the number of loops identified among the THP-1 down-sampled datasets, and found excellent agreement among the monocytic THP-1 (885 ± 8) and the macrophagic THP-1 (826 ± 17) down-sampled datasets (Supplementary Fig. S8, Supplementary Table 4). By contrast, we found that there are 451 and 997 loops in the primary monocytes and primary, GM-CSF-induced macrophages (Supplementary Table 4), respectively. Thus, there is a statistically significant difference in the number of loops between the primary and cell lines with both types of cells (*p* < 0.001, normality test), with a quite pronounced increase of nearly 2-fold in the number of loops in the monocytic THP-1 cells.

**Fig. 2:**
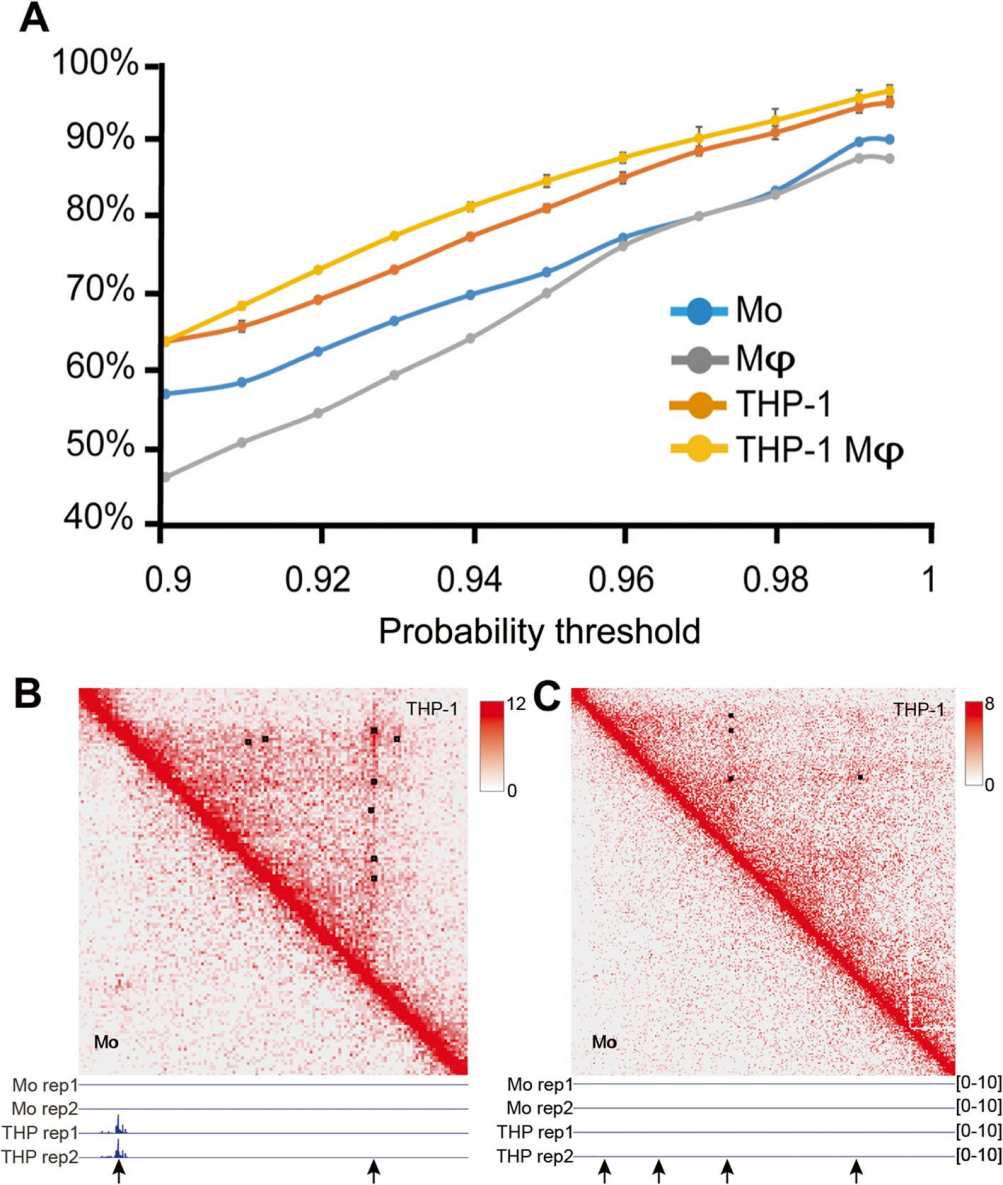
Differences in the structure at the loop level between primary and cell lines. **a** Relationship between the identical loop percentage and the probability threshold cutoff used to filter the loops, comparing all data to the full dataset of the corresponding THP-1 cells. **b** Hi-C heat map at Chr1:214.7-215.9 M (10 kb resolution) of the monocytic primary and THP-1 cells. The squared regions are the loops identified by Peakachu. The panel at the bottom shows the gene (CENPF) expression in this region, together with the loop anchor locations (arrowed) (**c**) Hi-C heat map at Chr2:33.8-36.2 M (10 kb resolution) of the monocytic primary and THP-1 cells. Annotations are the same as in (**b**)

We finally examined the similarity in the loop locations between the different datasets (Fig. 2, Supplementary Fig. S8). For this, we compared the extent to which the loops identified in each dataset (down-sampled THP-1 or primary cells) are at the same location as in the full THP-1 datasets. Using the threshold cutoff value of 0.97, the loops identified in the down-sampled THP-1 datasets were indeed highly similar to those in the full datasets, with 88.3 ± 0.6% and 89.9 ± 1.4% of loops identical in the monocytic and macrophagic cells, respectively. However, at the same threshold, there was significantly less (*p* < 0.001, normality test) identity of loop locations between the primary monocytes (80.1%) and the primary, GM-CSF-induced macrophage cells (80.1%) with the corresponding full THP-1 datasets (Fig. 2A). A similar level of difference between the down-sampled datasets and the primary cell datasets was observed over a broad range of threshold values (Fig. 2A), showing the robustness in this difference. Thus, in both the number and location of the loops, there are significant differences between the primary cells and the THP-1 cell line. Although the low sequencing depth of the primary cells prevents a comprehensive analysis of the relationship between loop changes and gene expression, we observed several instances of (apparent) changes at the loop-level that were associated with changes in expression as well as those that were not (Fig. 2b, c; Supplementary Fig. S9). A complete list of all differentially expressed genes between primary and cultured cells can be found in Supplementary Table 5.

## Conclusion

Although immortalized cell lines are unquestionably a staple of present biological research, it is also essential to understand the extent to which they faithfully reflect the properties of primary cells. Here, we show that there is a surprisingly large degree of differences in the chromatin structures of the THP-1 cell line and their primary cell counterparts at all levels of chromatin structure. The degree of differences in the compartments (~ 20%) indeed reflects a significant change in chromosome structure, especially among ostensibly highly related cells (that is, they are both monocytic cells), since at this level, most pairwise comparisons of different cell types reveal fewer differences [13]. Yet, in previous studies that identified this high degree of compartment-level differences, the structures at the TAD-level were nonetheless found to be nearly identical [18, 19]. However, we found that there was a drastic difference at the TAD level (19 and 48% different from the down-sampled datasets themselves) between these primary cells and the THP-1 cells, demonstrating that these cells are exceptionally different in chromatin structure at this level. Finally, even at the level of loops, we found that there are significant differences in both their number and location between these cells. With such wide-ranging differences in structure, it is perhaps not surprising that AP-1, which was found to play a significant role in the formation of loops during the trans-differentiation of THP-1 cells [8], is not up-regulated in the primary cells [9]. Thus, the critical components and molecular mechanisms underlying the structural changes in the primary cells during trans-differentiation remain to be discovered.

The functional significance of these structural differences between the THP-1 and the primary cells is evidenced by the clear correlation between the compartment and gene expression differences, with a significant enrichment of many different biological pathways in all different compartments, including those associated with immune-cell processes. We note that the lower sequencing depth of the primary cells prevents a comprehensive analysis of relationships between differences at the loop or TAD level and gene expression in the primary and THP-1 cells, although we did observe some differences in expression that appeared to be associated with significant differences in structure at the TAD level (Supplementary Fig. S10). In addition, the differences in the induction mechanisms (PMA versus GM-CSF) might have contributed to some of the differences observed between the macrophagic-type cells. Nonetheless, the differences observed between the primary monocytes and the THP-1 cells can only be owing to the transformation of the THP-1 cells from healthy cells to immortalized cells, whether during their transition to malignancy in the patient or subsequent to their isolation for culturing, or their evolution during prolonged culturing. Indeed, there is evidence for differences in chromatin structure between cancer cells and their healthy counterparts [21]. Moreover, for THP-1 cells in particular, there is early evidence for two different cell variants (one diploid and one aneuploidy [22]), as well as differences in Human Leukocyte Antigen alleles in current THP-1 cells from those described in the original cells [23]. Further, recently, THP-1 cells obtained from different biorepositories were shown to exhibit substantial genomic, transcriptomic, and proteomic differences, suggesting that these cells have undergone significant genetic drift following their isolation [24].

Thus, our results indicate that the THP-1 cell line has lost some of its primary monocytic features in chromatin structure, likely associated with changes in the activation of specific pathways, that lead to a differing phenotype. Hence, while immortalized cells are, without question, useful for analysis of biological processes within cells, there should be caution when attributing physiological significance to observations obtained with these cells that have not been directly validated with primary cells [19].

## Materials and methods

### Hi-C data processing

All Hi-C reads were mapped to the human hg19 reference genome iteratively using Bowtie2 as described in [25]. Reads mapped to multiple genomic locations and with low mapping quality (MAPQ < 30) were removed. Read pairs that mapped to the same restriction fragment and PCR duplicates were further removed. To eliminate the bias introduced by extreme read coverage, Hi-C reads mapped to the blacklist features defined in the ENCODE project were also removed [26]. Finally we generated Hi-C contact matrices at 10 kb, 40 kb, and 100 kb resolution and normalized using cooler [27].

### mRNA-Seq quality control and alignment of sequencing data

Raw reads were processed with Cutadapt (v1.18) [28] and Trimmomatic (v0.38) [29] to remove sequencing adapters, short reads (length < 35 bp) and low quality reads. FastQC was then used to ensure high quality reads [30]. The clean reads were mapped to the human genome (hg19) with the HISAT2 software [31] with same parameter in [32]. Gene expression levels were calculated based on TPM (transcript per kilobase million) with StringTie [31]. The GO functional enrichment analysis was performed with DAVID [33].

### Identification of compartments

Compartments were annotated using Juicer [10] at 200 kb resolution. The signs of the eigenvectors that indicate different types of compartments were determined according to the Pearson correlation coefficient (PCC) between the eigenvector and gene density as described in [34]. Eigenvectors are multiplied by − 1 if the PCC is negative. Positive eigenvectors indicate A-compartments and negative eigenvectors indicate B-compartments. To validate the compartments annotated by Juicer, we recalculated the eigenvectors of the Hi-C matrix using cworld [35]. Overall, we found a very high agreement in the compartment-level analysis between these two methods (Supplementary Fig. S4 and S11): ~ 95% of the compartment locations are the same, and the Pearson correlation between the eigenvalues is greater than 0.9. Moreover, both software show a similar amount of differences in compartment-level interactions between the primary monocytes and monocytic THP-1 cells (20 and 21%, respectively).

### Identification of TADs and loops

The identification of TADs is based on the 40 kB resolution normalized matrix, which is the highest resolution the our dataset could achieve [36]. TADs were annotated using the TopDom R package [37] with a window size of 20, which is within the recommended range (5–20). All loops were annotated in the 10 kb resolution normalized Hi-C matrix. The loops were annotated using Peakachu, which has been shown to be a reliable loop caller and is robust to sequencing depth [20]. For all loop predictions in the work, the cutoff threshold was 0.97 as recommended by the authors of this software.

### The comparison of similarity of loop locations

The loop anchors were defined in the 10 kb resolution map at either the start or end points of the loops. Due to the sparsity introduced by down-sampling from the full dataset, the loop center in the down-sampled dataset may have shifted 1 to 2 bins (each bin is 10 kb) compared to the loop centers in full dataset. Thus, identical loops in the different datasets were defined as those loops with both anchors within 20 kb of each other. The “identical percentage” is the number of identical loops divided by the number of all loops in the down-sampled or primary cell datasets.

## Supplementary Information

**Additional file 1.**

**Additional file 2.**

**Additional file 3.**

**Additional file 4.**

**Additional file 5.**

**Additional file 6 **: **Supplementary Figure S1**. Comparison of gene expression levels in different compartments. **Supplementary Figure S2**. Similarities/differences of compartments between primary cells and THP-1 cells**. Supplementary Figure S3**. Example of differentially expressed genes whose changed expression is associated with the changes of compartment. **Supplementary Figure S4.** Compartments identified with different software yield similar results at loci of immune-related genes. **Supplementary Figure S5.** Comparison of gene expression levels at regions that switched compartments. **Supplementary Figure S6.** Relationship between gene expression levels and compartment changes in primary, GM-CSF-induced macrophages and macrophagic THP-1 cells. **Supplementary Figure S7.** Similarity of TAD locations between the primary and down-sampled THP-1 datasets. **Supplementary Figure S8.** Numbers of loops in the down-sampled datasets in the monocytic and macrophagic THP-1 cells**. Supplementary Figure S9.** Hi-C heatmap showing loop-scale chromosomal structural differences. **Supplementary Figure S10.** Correlation between chromosomal structural changes and gene expression. **Supplementary Figure S11.** A/B compartments identified by different software exhibit high congruence.

## Data Availability

The data of the THP-1 cells were obtained from the Gene Expression Omnibus (GEO) with series number GSE96800 (RNA-seq) and Sequencing Read Archive (SRA) with accession number PRJNA385337 (Hi-C). The data of the primary cells were obtained from ArrayExpress with following codes: E-MTAB-8261 (RNA-seq) and E-MTAB-8262 (Hi-C).
