# Supplementary material for: Monocytic THP-1 cells diverge significantly from their primary counterparts: a comparative examination of the chromosomal conformations and transcriptomes
Source: Hereditas. 2021 Nov 5;158:43. doi: 10.1186/s41065-021-00205-w (PMC8569982; doi:10.1186/s41065-021-00205-w)
Supplement: Supplementary file 6 — Additional file 6 : Supplementary Figure S1. Comparison of gene expression levels in different compartments. Supplementary Figure S2. Similarities/differences of compartments between primary cells and THP-1 cells. Supplementary Figure S3. Example of differentially expressed genes whose changed expression is associated with the changes of compartment. Supplementary Figure S4. Compartments identified with different software yield similar results at loci of immune-related genes. Supplementary Figure S5. Comparison of gene expression levels at regions that switched compartments. Supplementary Figure S6. Relationship between gene expression levels and compartment changes in primary, GM-CSF-induced macrophages and macrophagic THP-1 cells. Supplementary Figure S7. Similarity of TAD locations between the primary and down-sampled THP-1 datasets. Supplementary Figure S8. Numbers of loops in the down-sampled datasets in the monocytic and macrophagic THP-1 cells. Supplementary Figure S9. Hi-C heatmap showing loop-scale chromosomal structural differences. Supplementary Figure S10. Correlation between chromosomal structural changes and gene expression. Supplementary Figure S11. A/B compartments identified by different software exhibit high congruence. [file 41065_2021_205_MOESM6_ESM.doc]

**Supplementary Information**

**Monocytic THP-1 cells diverge significantly from their primary counterparts: a comparative examination of the chromosomal conformations and transcriptomes**

Yulong Liu, Hua Li, Daniel M. Czajkowsky^*^ and Zhifeng Shao

**Supplementary Fig. S1. Comparison of gene expression levels in different compartments.**

**Supplementary Fig. S2. Similarities/differences of compartments between primary cells and THP-1 cells.**

**Supplementary Fig. S3. Example of differentially expressed genes whose changed expression is associated with the changes of compartment.**

**Supplementary Fig. S4. Compartments identified with different software yield similar results at loci of immune-related genes.**

**Supplementary Fig. S5. Comparison of gene expression levels at regions that switched compartments.**

**Supplementary Fig. S6. Relationship between gene expression levels and compartment changes in primary, GM-CSF-induced macrophages and macrophagic THP-1 cells.**

**Supplementary Fig. S7. Similarity of TAD locations between the primary and down-sampled THP-1 datasets.**

**Supplementary Fig. S8. Numbers of loops in the down-sampled datasets in the monocytic and macrophagic THP-1 cells.**

**Supplementary Fig. S9. Hi-C heatmap showing loop-scale chromosomal structural differences.**

**Supplementary Fig. S10. Correlation between chromosomal structural changes and gene expression.**

**Supplementary Fig. S11. A/B compartments identified by different software exhibit high congruence.**


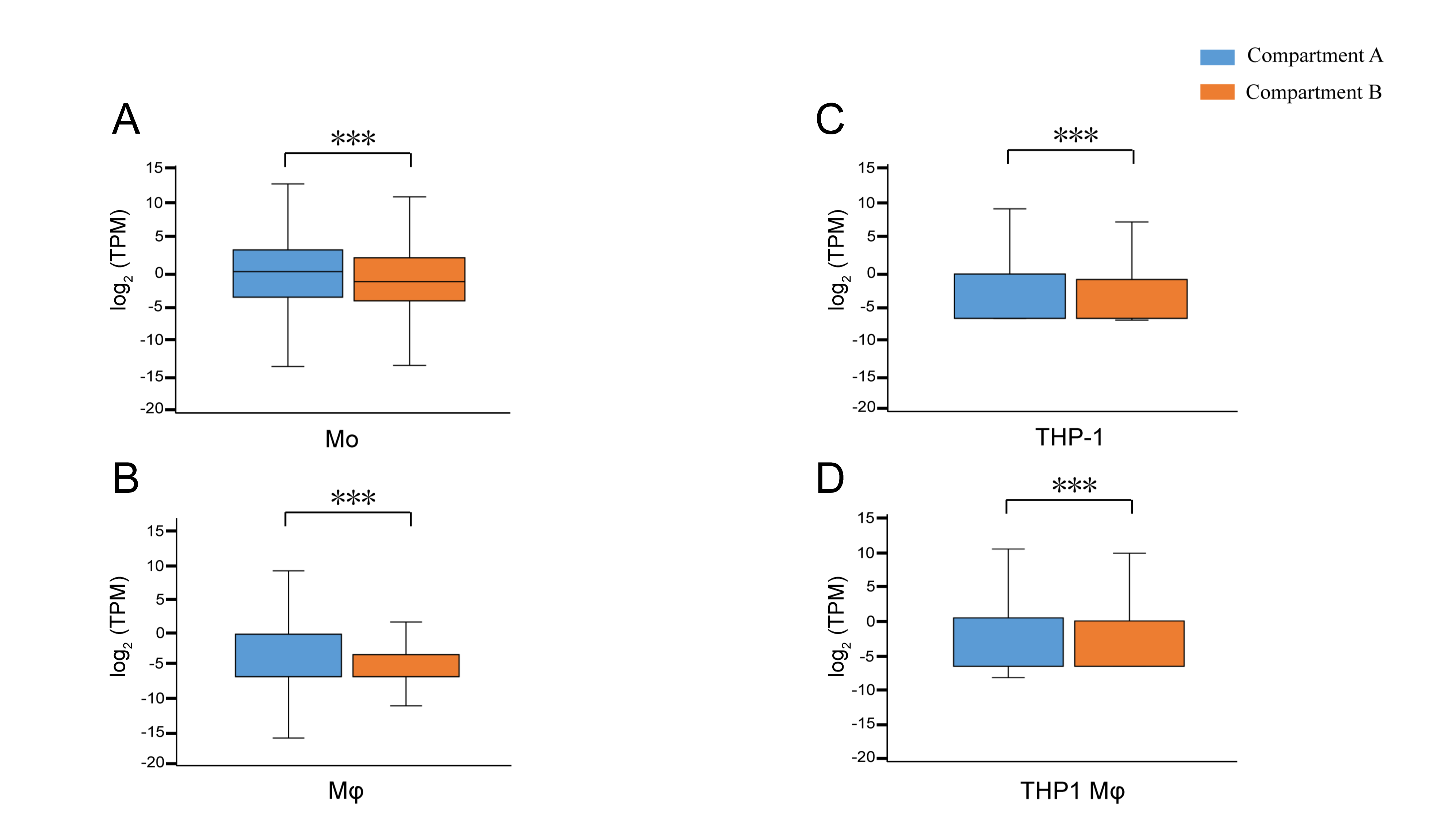


**Supplementary Fig. S1. Comparison of gene expression levels in different compartments.**

As expected, the gene expression in the A-compartments is greater than in the B-compartments (*p* < 0.001, Wilcoxon rank sum test) in (A) primary monocytes, (B) primary, GM-CSF-induced macrophages, (C) monocytic THP-1 cells, and (D) macrophagic THP-1 cells.

**
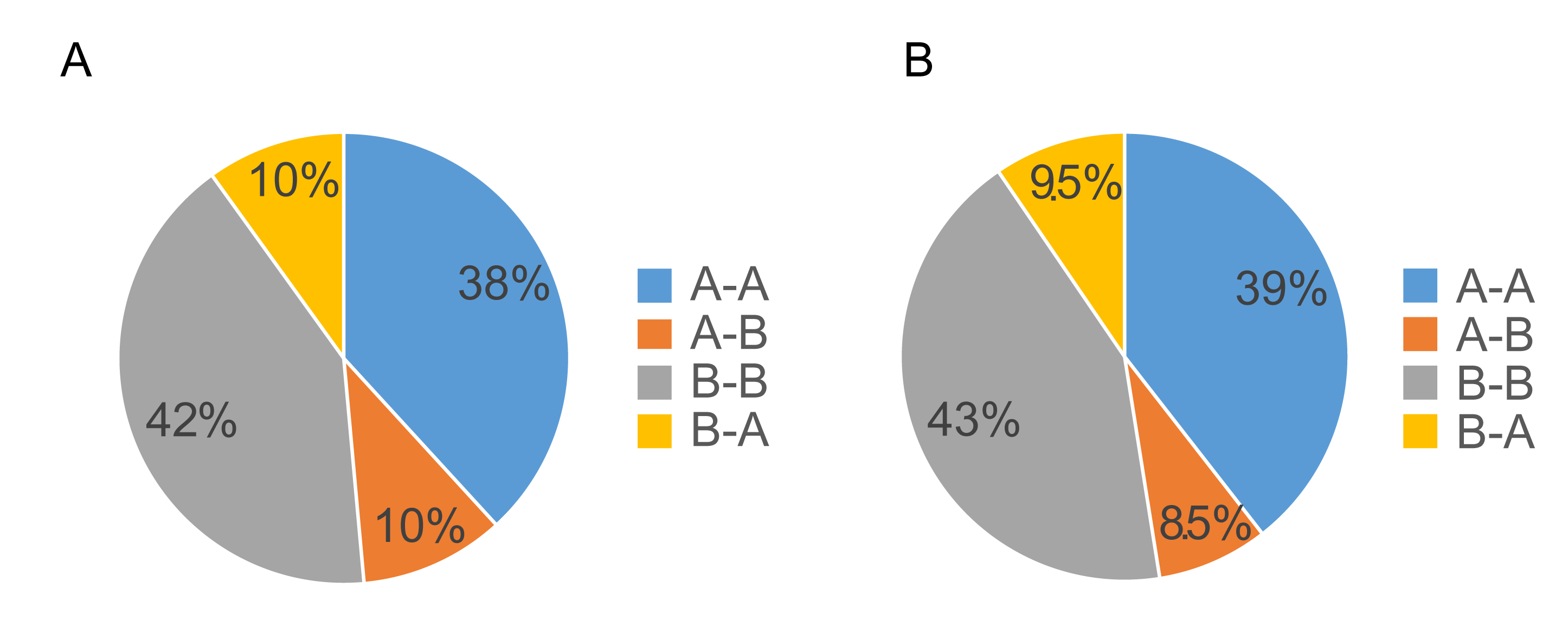
**

**Supplementary Fig. S2. Similarities/differences of compartments between primary cells and THP-1 cells.**

Comparison of compartments between (A) primary monocytes and THP-1 cells and (B) primary, GM-CSF-induced macrophages and macrophagic THP-1 cells.


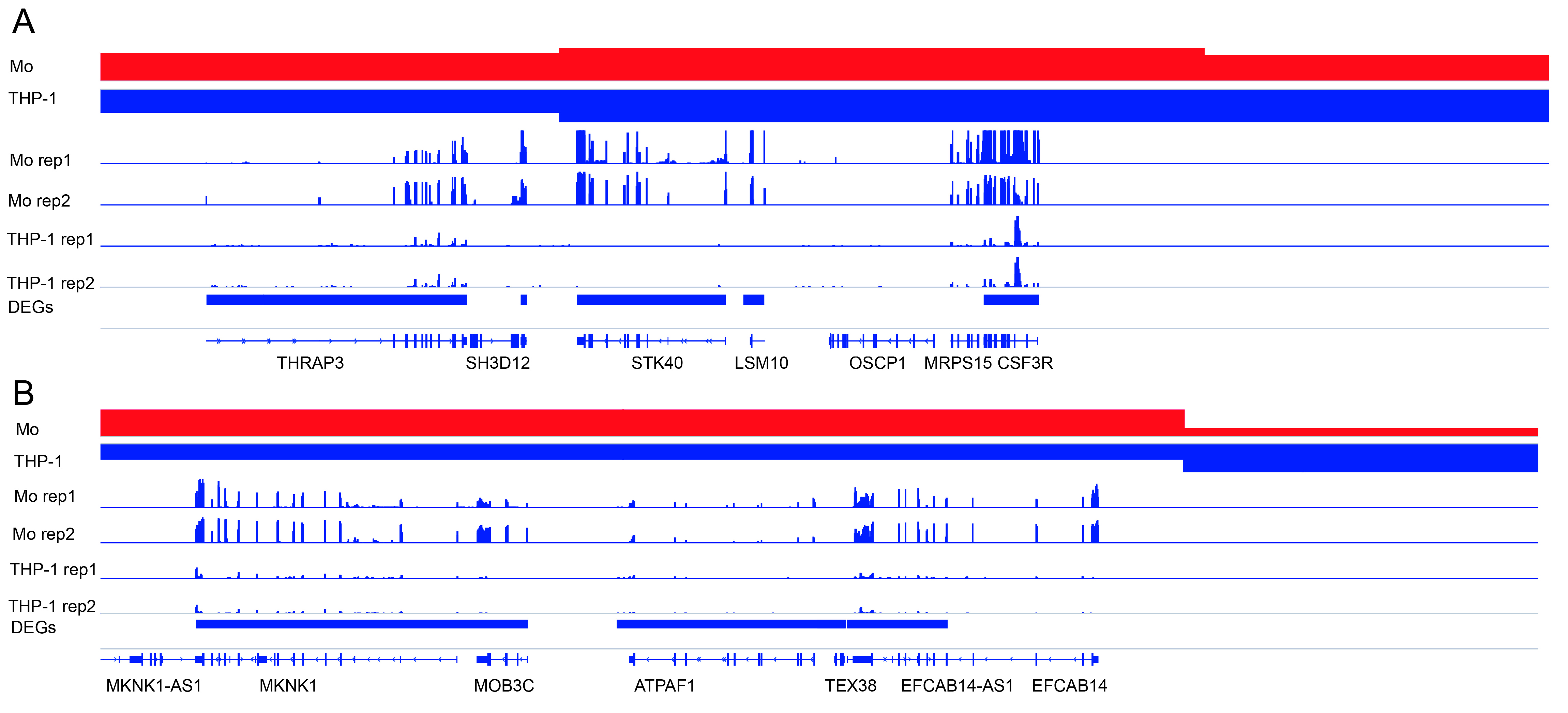


**Supplementary Fig. S3. Example of differentially expressed genes whose changed expression is associated with a change of compartment.**

(A) Shown in the top panel is the compartment annotation at Chr1:36.66-37.109M, with A-compartment colored red and B-compartment colored blue. The bottom panel shows the expression of the genes in this region, together with an indication of those genes identified as differentially expressed (DEG). (B) Similar to (A) but at Chr1:47-47.27M.


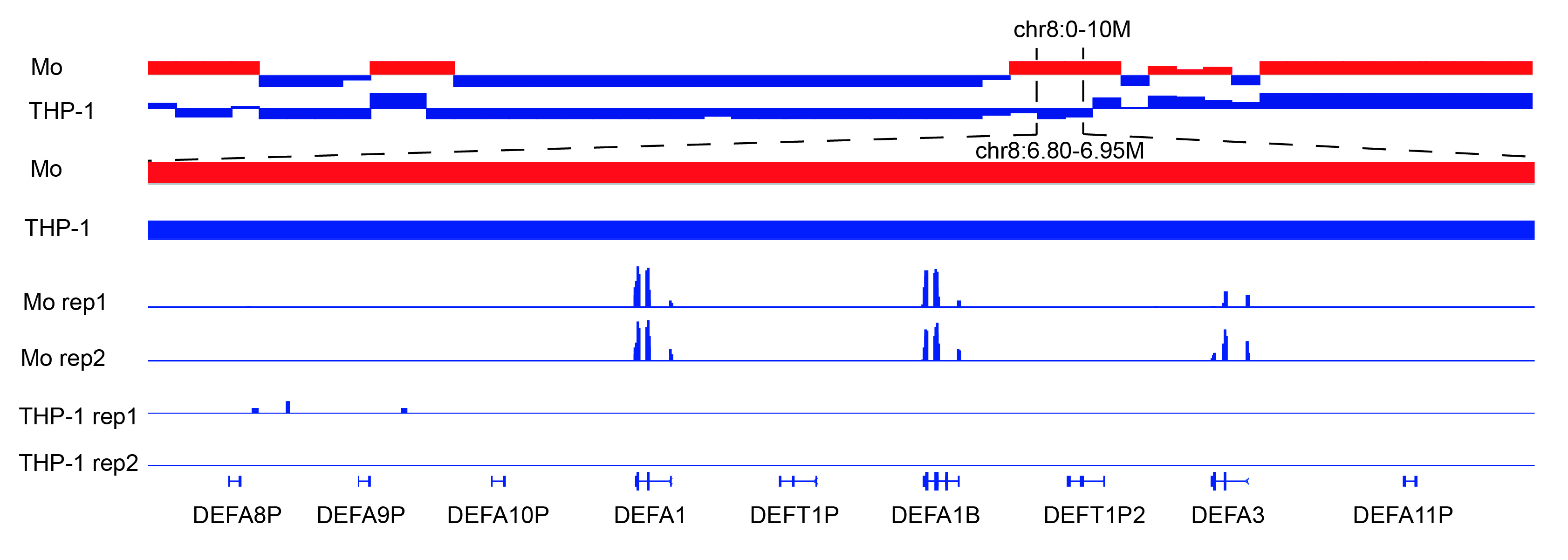


**Supplementary Fig. S4. Compartments identified with different software yield similar results at loci of immune-related genes.**

This is the same genomic region as shown in Fig 1c. Here, the compartments identified with cworld are shown while those in Fig 1c were obtained with Juicer. The compartments are colored as in Fig 1c.


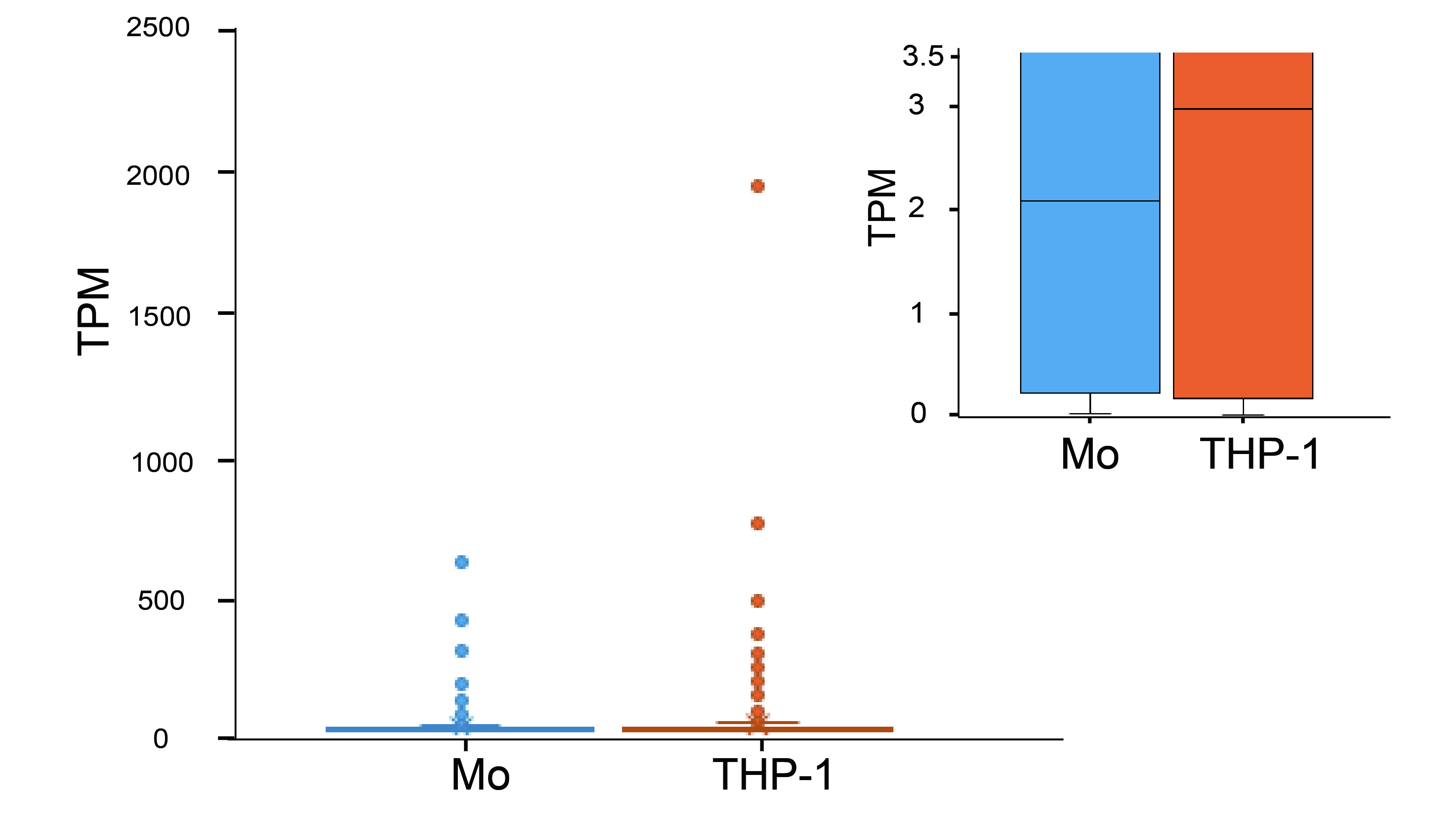


**Supplementary Fig. S5. Comparison of gene expression levels at regions that switched compartments.** This is a similar plot as Fig 1d but with the y-axis as TPM and not log_2_(TPM) as in Fig 1d. The inset in the upper right is the zoomed-in region near the mean expression value.


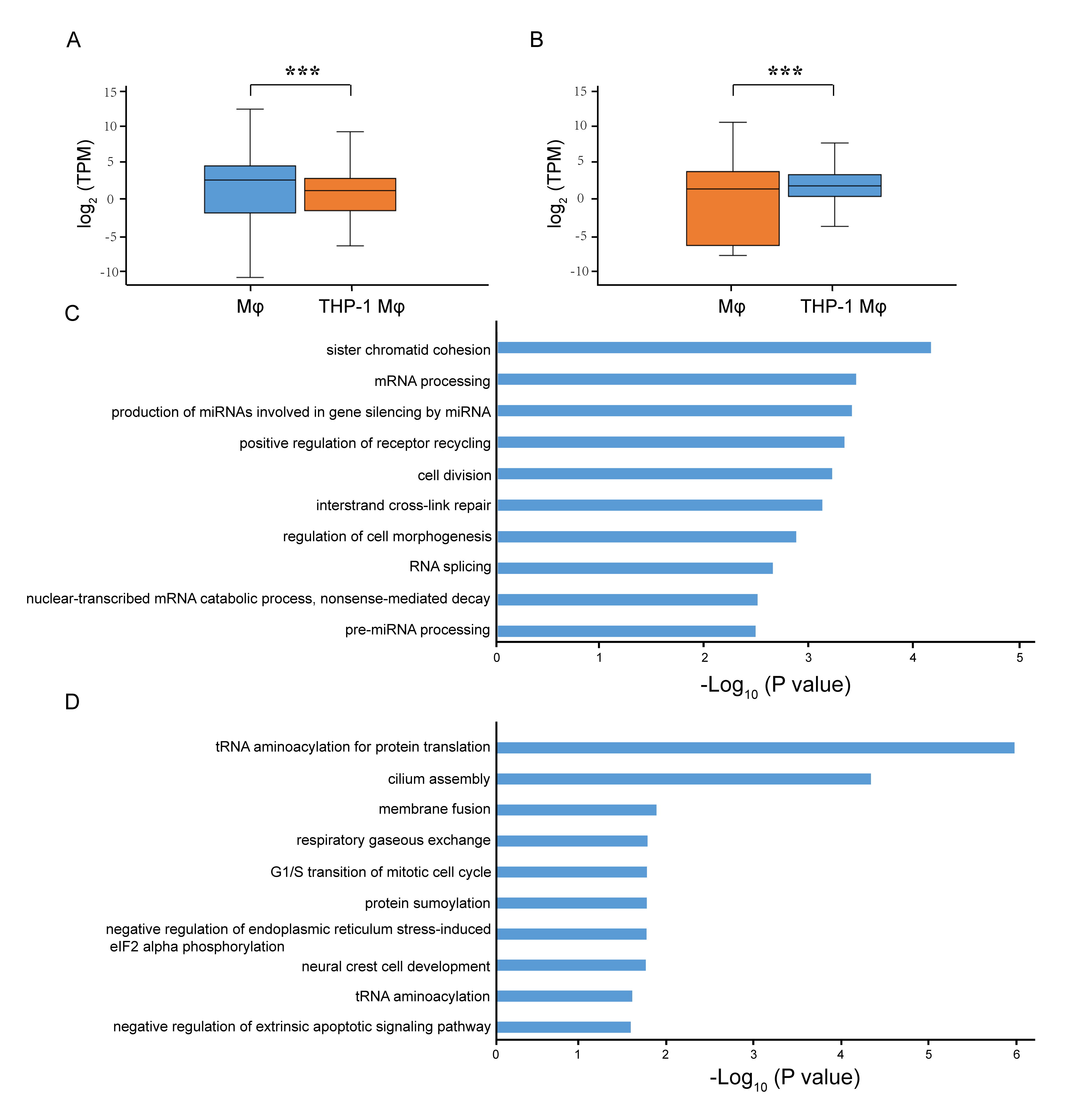


**Supplementary Fig. S6. Relationship between gene expression levels and compartment changes in primary, GM-CSF-induced macrophages and macrophagic THP-1 cells.**

(A) Comparison of gene expression levels at regions that switched from primary, GM-CSF-induced macrophage A-compartment to THP-1 B compartment (“***” represents *p* < 0.001, Wilcoxon rank sum test; TPM refers to transcript per million). (B) Comparison of gene expression levels at regions that switched from primary, GM-CSF-induced macrophage B compartment to macrophagic THP-1 A-compartment. (C) Gene ontology analysis of genes that reside in regions that switched from primary, GM-CSF-induced macrophage A-compartment to macrophagic THP-1 B-compartment. (D) Gene ontology analysis of genes that reside in regions that switched from primary, GM-CSF-induced macrophage B-compartment to macrophagic THP-1 A-compartment.

**
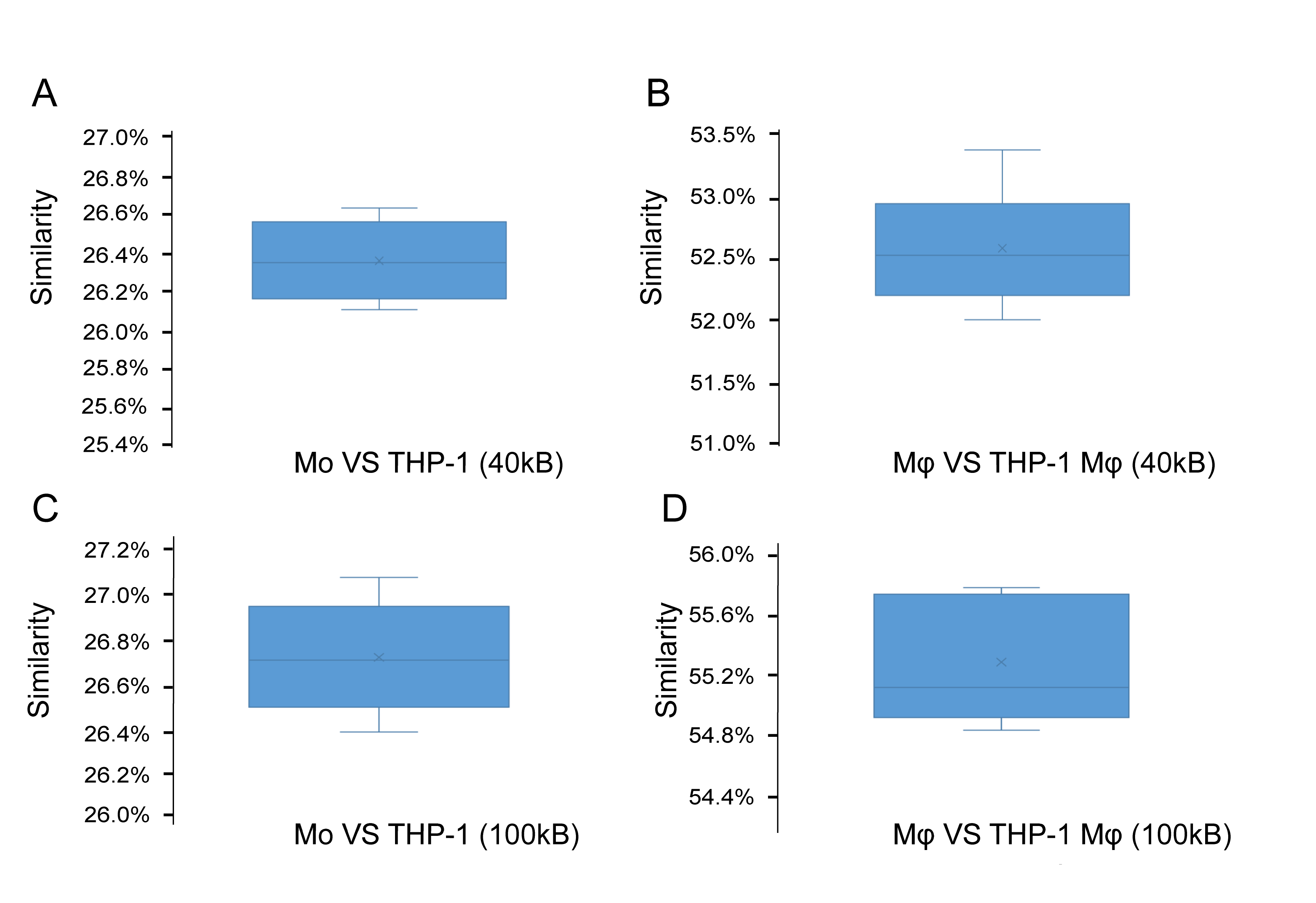
**

**Supplementary Fig. S7. Similarity of TAD locations between the primary and down-sampled THP-1 datasets.**

Box plots showing the similarity of the TAD locations between each of the five down-sampled THP-1 datasets and the primary cells for the (A) monocytic cells at 40 kB resolution, (B) macrophagic cells at 40 kB resolution, (C) monocytic cells at 100 kB resolution, and (D) macrophagic cells at 100 kB resolution.

**
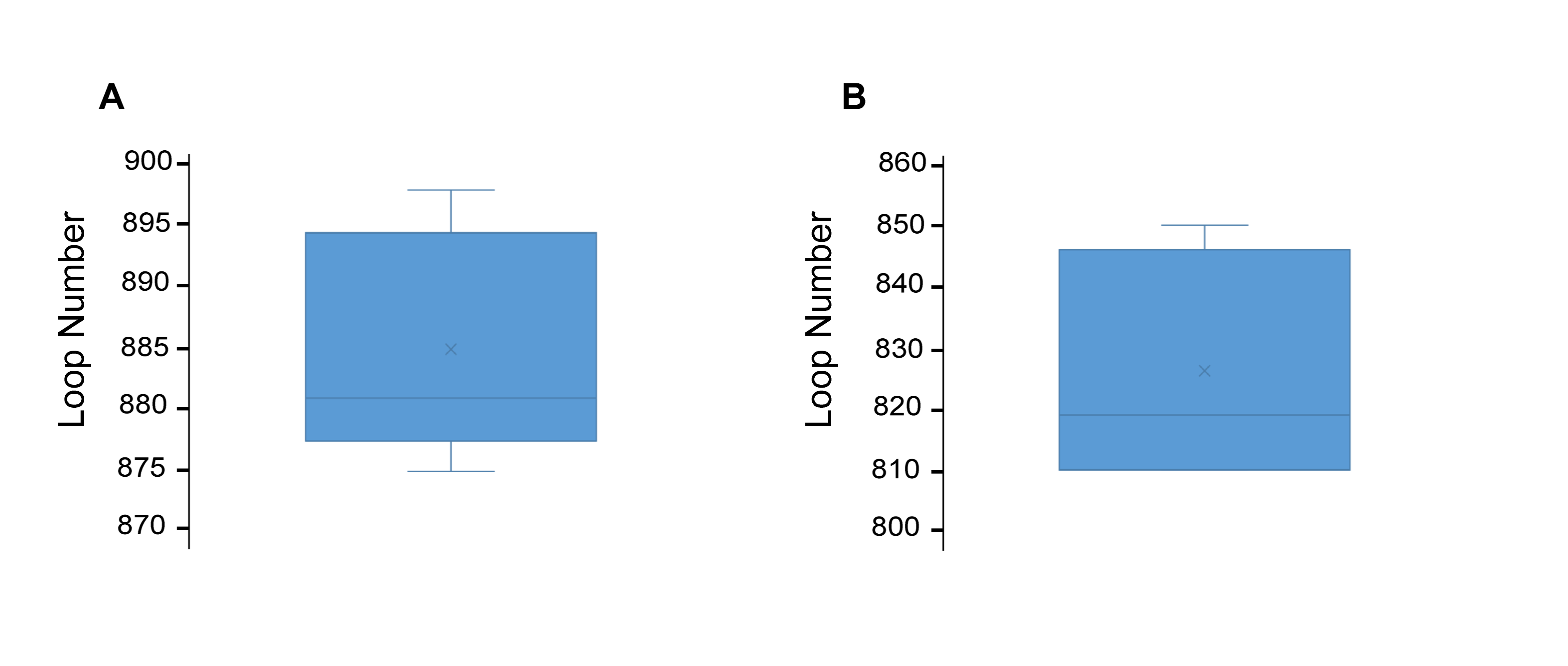
**

**Supplementary Fig. S8. Numbers of loops in the down-sampled datasets in the monocytic and macrophagic THP-1 cells.**

Box plot of the numbers of loops in the five THP-1 down-sampled datasets for the (A) monocytic and (B) macrophagic cells.


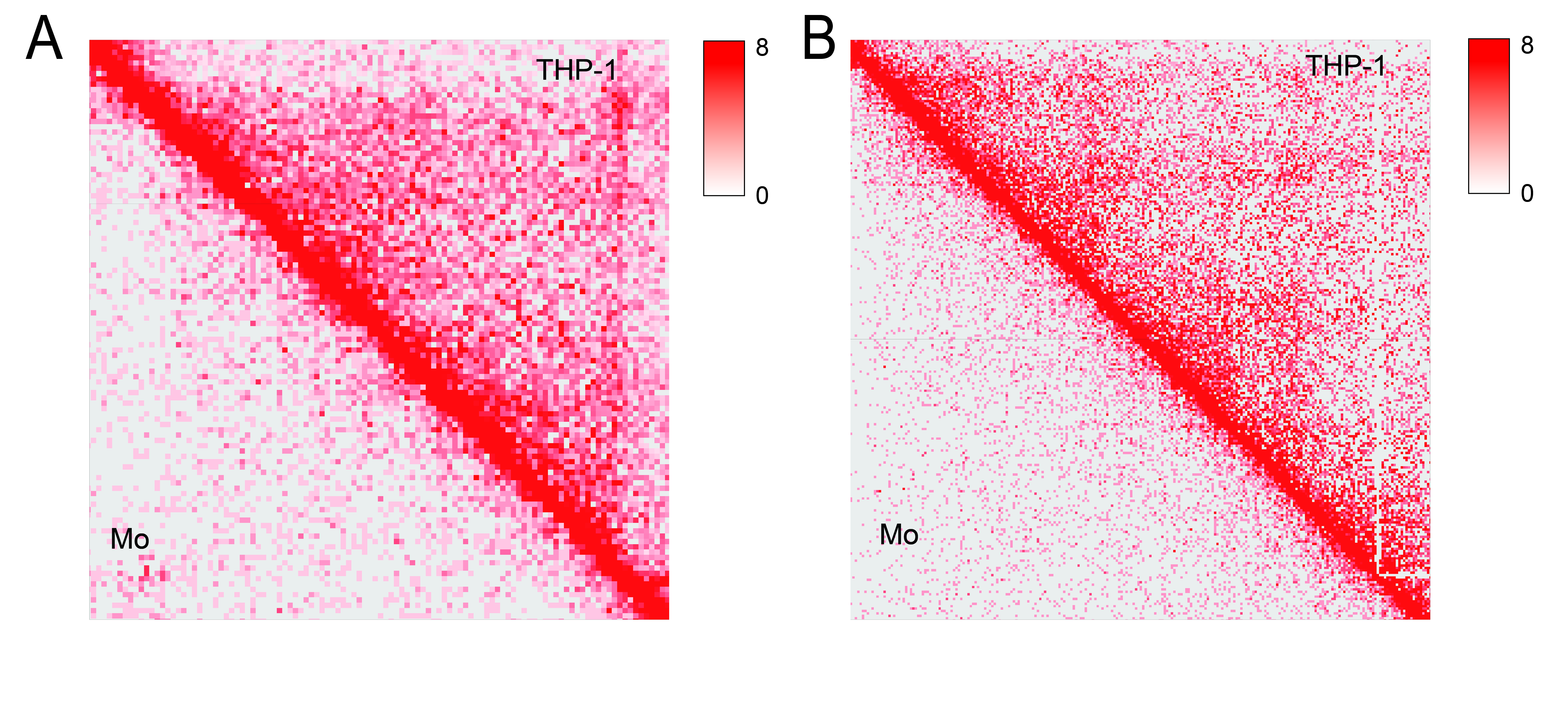


**Supplementary Fig. S9. Hi-C heatmap showing loop-scale chromosomal structural differences.**

Identical image as Figure 2b, c but without the boxed regions to enable inspection of the bare heatmap.


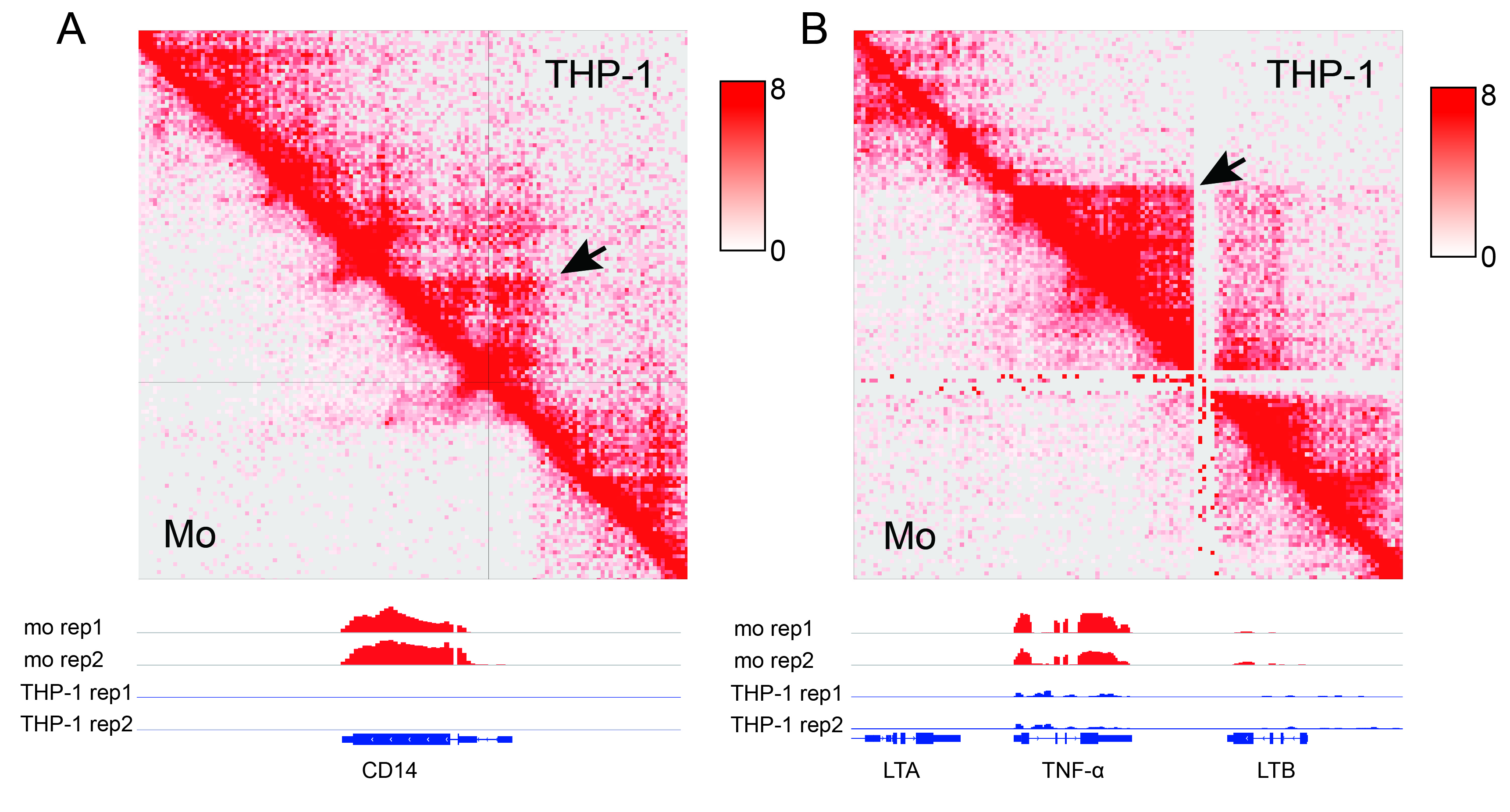


**Supplementary Fig. S10. Correlation between chromosomal structural changes and gene expression.**

(A) Hi-C heat map at Chr5:139.1-140.5M (10 kb resolution) of the monocytic primary and THP-1 cells. The arrow points to the location of a TAD in both primary monocyte and THP-1 cells that overlaps the CD14 gene. The interaction strength within this TAD in the primary monocyte is much lower than that in the THP-1 cells, consistent with a less compact structure. Shown at the bottom is the gene expression of CD14 in these cells, which agrees with previous reports^38^. (B) Hi-C heat map at Chr6:31.1-32.4M (10 kb resolution) of the monocytic primary and THP-1 cells. The arrow points to a TAD that overlaps the TNF-α gene. The interaction strength within this TAD is also much lower in the primary monocyte than in the THP-1 cells. The bottom panel shows the expression of TNF-α, in these cells, also in agreement with previous reports^39,40^.


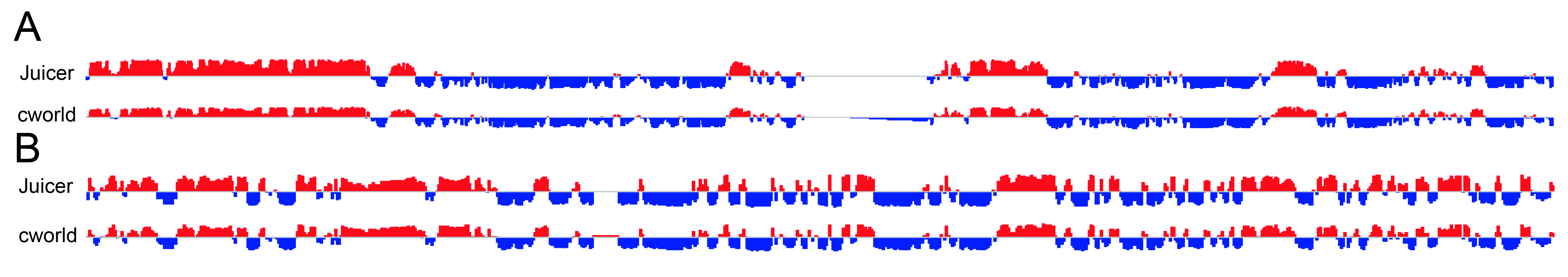


**Supplementary Fig. S11. A/B compartments identified by different software exhibit high congruence.**

Overall, ~95% of the compartment locations annotated using Juicer and cworld are the same, and the eigenvalues obtained by the two software are highly correlated (Pearson correlation, R > 0.9). This high agreement is also evident by inspection of specific annotated regions, such as chromosome 1 (A) or chromosome 6 (B) of the primary monocyte.
